# *TANGO2*: expanding the clinical phenotype and spectrum of pathogenic variants

**DOI:** 10.1038/s41436-018-0137-y

**Published:** 2018-09-24

**Authors:** Jennifer N. Dines, Katie Golden-Grant, Amy LaCroix, Alison M. Muir, Dianne Laboy Cintrón, Kirsty McWalter, Megan T. Cho, Angela Sun, J. Lawrence Merritt, Jenny Thies, Dmitriy Niyazov, Barbara Burton, Katherine Kim, Leah Fleming, Rachel Westman, Peter Karachunski, Joline Dalton, Alice Basinger, Can Ficicioglu, Ingo Helbig, Manuela Pendziwiat, Hiltrud Muhle, Katherine L. Helbig, Almuth Caliebe, René Santer, Kolja Becker, Sharon Suchy, Ganka Douglas, Francisca Millan, Amber Begtrup, Kristin G. Monaghan, Heather C. Mefford

**Affiliations:** 1 Department of Medicine, Division of Medical Genetics, University of Washington, Seattle, Washington USA; 2 Division of Genetic Medicine, Seattle Children’s Hospital, Seattle, Washington USA; 3 Department of Pediatrics, University of Washington, Seattle, Washington USA; 4 GeneDx, Gaithersburg, Maryland USA; 5 Division of Medical Genetics, Ochsner Health System and University of Queensland, Brisbane, Australia; 6 Ann & Robert H. Lurie Children’s Hospital of Chicago, Northwestern University Feinberg SOM, Chicago, Illinois USA; 7 Genetics and Metabolic Clinic, St. Luke’s Children’s Hospital System, Boise, Idaho USA; 8 Department of Neurology, University of Minnesota, Minneapolis, Minnesota USA; 9 Cook’s Children Genetic Center, Fort Worth, Texas USA; 10 Division of Neurology, Children’s Hospital of Philadelphia, Philadelphia, Pennsylvania USA; 11 Department of Neuropediatrics, Universitätsklinikum Schleswig Holstein Campus Kiel, Kiel, Germany; 12 Institute for Human Genetics, Universitätsklinikum Schleswig Holstein Campus Kiel, Kiel, Germany; 13 Department of Pediatrics, University Medical Center Eppendorf, Hamburg, Germany

**Keywords:** developmental delay DNA copy-number variation, epilepsy, intragenic deletion, exome sequencing

## Abstract

**Purpose:**

*TANGO2*-related disorders were first described in 2016 and prior to this publication, only 15 individuals with *TANGO2*-related disorder were described in the literature. Primary features include metabolic crisis with rhabdomyolysis, encephalopathy, intellectual disability, seizures, and cardiac arrhythmias. We assess whether genotype and phenotype of *TANGO2*-related disorder has expanded since the initial discovery and determine the efficacy of exome sequencing (ES) as a diagnostic tool for detecting variants.

**Methods:**

We present a series of 14 individuals from 11 unrelated families with complex medical and developmental histories, in whom ES or microarray identified compound heterozygous or homozygous variants in *TANGO2*.

**Results:**

The initial presentation of patients with *TANGO2*-related disorders can be variable, including primarily neurological presentations. We expand the phenotype and genotype for *TANGO2*, highlighting the variability of the disorder.

**Conclusion:**

*TANGO2*-related disorders can have a more diverse clinical presentation than previously anticipated. We illustrate the utility of routine ES data reanalysis whereby discovery of novel disease genes can lead to a diagnosis in previously unsolved cases and the need for additional copy-number variation analysis when ES is performed.

## Introduction

*TANGO2* (transport and Golgi organization 2 homolog) was first identified in 2016 and encodes a protein that is postulated to be involved in redistributing the Golgi membranes into the endoplasmic reticulum (ER). As previously reported, exome sequencing (ES) has identified pathogenic *TANGO2* variants in 15 individuals presenting in metabolic crisis with rhabdomyolysis, seizures, progressive intellectual disability, encephalopathy, and cardiac arrhythmias.^1,2^ As a newly recognized disorder, additional clinical and molecular information is necessary for clinicians and families to recognize and understand the phenotypic spectrum of the disorder. In this collaborative case series, we present clinical and molecular data for 14 previously unreported individuals from 11 unrelated families who harbor compound heterozygous or homozygous *TANGO2* variants, expanding the molecular profile and clinical phenotype of *TANGO2*-related disorder.

## Materials and methods

Individuals were initially identified by clinical exome sequencing, gene panel, and/or chromosome microarray that detected pathogenic variants in *TANGO2*. Families 1–8 underwent ES along with analysis using an in-house copy-number variant (CNV) detection algorithm^3^ (GeneDx, Gaithersburg, MD). Family 8 had chromosome microarray testing, followed by ES analysis limited to a specific phenotype-driven gene list provided by the ordering clinician that included *TANGO2*. Family 9 had chromosome microarray testing, followed by reanalysis of exome data (Ambry Genetics, Santa Clara, CA). Family 10 had a chromosome microarray at Kiel (Human Genetics). Family 11 had a chromosome microarray at Kiel (Human Genetics), Germany and ES in Cologne, Germany. This study was approved by the Institutional Review Board at the University of Washington. Informed consent was obtained from all affected individuals or their guardians per the specifications of the respective home institutions, including consent to publish photographs for families 1 and 8. The population frequency of each variant is listed in Table S1.

### RNA sequencing

RNA was extracted from patient 1 and control fibroblast cell lines using Trizol (Ambion) and RNA Clean and Concentrator (Zymo Research). RNA sequencing library preparation was performed following the manufacturer's instructions using the TruSeq RNA Library Prep Kit v2 (Illumina), which captures non-strand-specific messenger RNA (mRNA) libraries via poly-A selection. Paired-end 100-bp sequencing was performed on an Illumina HiSeq 4000 with targeted sequencing coverage of 50 M reads. Raw reads were mapped to the human genome (GRCh37/UCSC hg19) using STAR (v.2.2.1). The region surrounding the putative splice site variant NM_152906.6:c.711-3C>G was manually evaluated using the Integrative Genomics Viewer. Sashimi plots were generated using MISO (v.0.5.4).

Patient 1 and control RNA was reverse transcribed following the manufacturer’s instructions using oligo(dT) nucleotides and SuperScript II (Invitrogen). Polymerase chain reaction (PCR) to verify splicing defects was performed using primers designed to amplify regions of *TANGO2* encompassing exon 5 through exon 9, and exon 5 through intron 7. GAPDH was used as a loading control. Primer sequences are listed in Table S2.

## Results

**Fig. 1:**
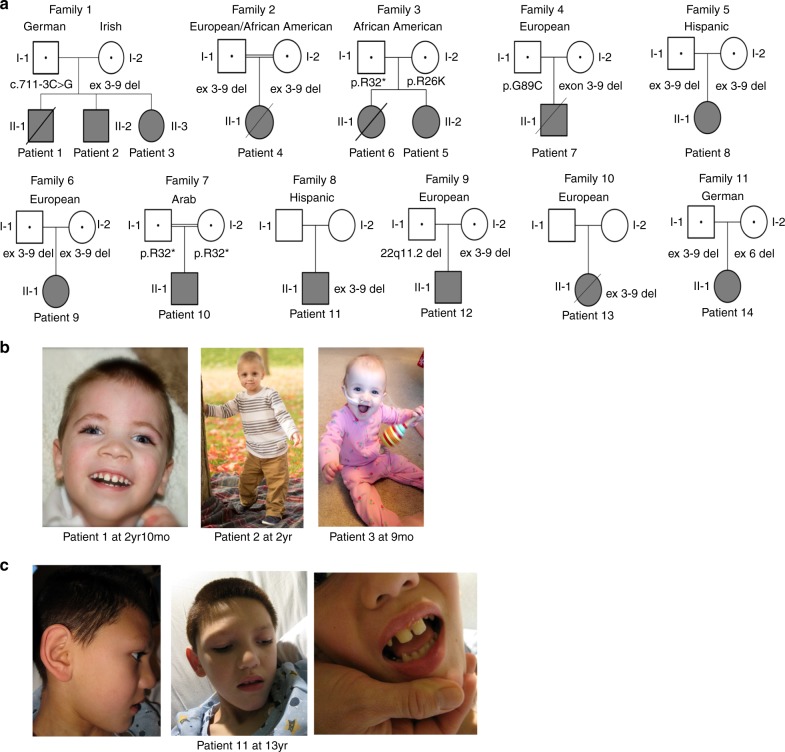
Pedigrees with variant segregation data (if known) and patient photos. **a** Pedigrees from 11 families. **b** Patient photos from family 1 including patient 1 at 2 years 10 months, patient 2 at 2 years, and patient 3 at 9 months. **c** Patient photos from patient 11 at 13 years.

We describe the clinical and molecular details of 14 individuals with compound heterozygous or homozygous pathogenic variants in *TANGO2* from 11 unrelated families (Table 1, Fig. 1a, Table S3). Demographics include 8 females and 6 males with ages ranging from 8 months to 26 years old. Self-declared ancestry included 8/14 of European descent, 2/14 of African American ancestry, 2/14 of Latino ethnicity, 1/14 of European/African American ancestry, and 1/14 of Arab ancestry. In two families, parents were consanguineous.

**Table 1:** Summary of clinical findings of our patients in comparison to prior cases

	P1	P2	P3	P4	P5	P6	P7	P8	P9	P10	P11	P12	P13	P14	Sum (%)	Lit^1, 2^ (%)
Paternal allele	c.711-3C>G	c.711-3C>G	c.711-3C>G	Ex 3–9 del	c.94C>T p.R32*	c.94C>T p.R32*	c.265G>T p.G89C	Ex 3–9 del	Ex 3–9 del	c.94C>T p.R32*	Ex 3–9 del^	22q11.2 del	Ex 3–9 del^	Ex 3–9 del	Fig. 4	Fig. 4
Maternal allele	Ex 3–9 del	Ex 3–9 del	Ex 3–9 del	Ex 3–9 del	c.77G>A p.R26K	c.77G>A p.R26K	Ex 3–9 del	Ex 3–9 del	Ex 3–9 del	c.94C>T p.R32*	Ex 3-9 del^	Ex 3–9 del	Ex 3-9 del^	Ex 6 del	Fig. 4	Fig. 4
Family	1	1	1	2	3	3	4	5	6	7	8	9	10	11		
Age of onset	5 m	9 m	8 m	1 yr	18 m	27 m	4 m	18 m	6 m	2 yr	6 m	9 m	6 m	1 yr	4–27 m	3.5 m–8 yr
Initial symptoms	DD	DD	DD	DD	DD/HG GTC	MC	MC	FS	DD	Ataxia	DD	DD	DD	DD	DD 9/14 Sz 2/14 MC 3/14 UG 1/14	DD 9/15 Sz 2/15 MC 3/15 UG 1/15
**Cardiac manifestations**																
Arrhythmia	-	-	-	+^g^	+^h^	-	+^i^	-	-	-	-	+^j^	+^c^	-	5/14 (36)	10/15 (67)
SCA	-	-	-	+	-	+	-	-	-	-	-	-	+	-	3/14 (21)	2/15 (13)
Pacemaker	-	-	-	+	-	-	-	-	-	-	-	-	-	-	1/14 (7)	4/15 (27)
ECHO	LVH	ND	N	↓ fx	DCM, ↓ fx	DCM	Mild ↓ fx	PT	N	N	N	N	N	N	6/14 (43)	2/15 (13)
**Neurologic findings**																
Seizures	+	+	+	+	+	-	+	+	-	+	-	-	+	+	10/14 (71)	12/15 (80)
Sz type /EEG	Mult. types^a^/EE	GTC	Mult. types^b^/EE	Partial complex	HG GTC	n/a	Multifocal, IS	GTC	n/a	HG GTC	EE	n/a	GTC	HG GTC, SE		
Refractory	+	-	+	-	-	n/a	+	-	n/a	-	n/a	n/a	-	-	3/14 (21)	1/3 (33)

*^*, parental testing not done; *+*, present; *-*, absent; *d.*, deceased (age at death); *DCM*, dilated cardiomyopathy; *DD*, developmental delay; *EE*, epileptic encephalopathy; *EEG*, electroencephalopgraphy; *FS*, febrile seizures; *fx*, function; *GT*, gastrostomy tube; *GTC*, generalized tonic–clonic; *HG GTC*, hypoglycemic generalized tonic–clonic; *IS*, infantile spasms; *Lit (%)*, summary of individuals from literature (Lalani et al.; Kremer et al.); *LVH*, left ventricular hypertrophy; *MC*, metabolic crisis; *N*, normal; *ND*, not done; *P#*, patient #; *PM*, postmortem; *PT*, prominent trabeculations; *SE*, status epilepticus; *Sum (%)*, summary of individuals from our case series; *Sz,* seizure; *TdP*, torsades de pointes, *U*, unknown; *UG*, unsteady gait; *VF*, ventricular fibrillation; *VT*, ventricular tachycardia; *w/a*, with assistance

^a^Infantile spasms, myoclonic, tonic, multifocal–bifrontal, and right parietal.

^b^Myoclonic, myoclonic–astatic, generalized tonic–clonic/EEG findings including epileptic encephalopathy and multifocal epileptiform discharges maximal in the bicentral region.

**Fig. 2:**
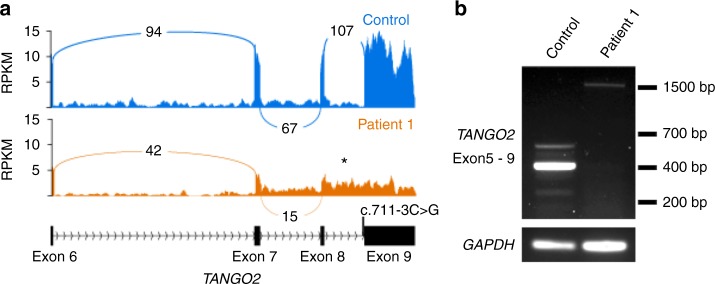
**NM_152906.6:c.711-3C>G causes aberrant splicing of** ***TANGO2***. **a** Sashimi plot showing *TANGO2* transcripts isolated form patient 1 and control fibroblasts. Intron 8 is retained in patient 1 transcripts (*). **b** Semiquantitative reverse transcription polymerase chain reaction (RT-PCR) validation of aberrant splicing of *TANGO2* in patient 1. Primers were designed to amplify exons 5 through exon 9 of *TANGO2*. The expected product size for a fully spliced transcript is 395 bp, while the expected product size if intron 8 was retained is 1499 bp.

The most common pathogenic variant in our cohort is the previously described^1,2^ deletion that encompasses exons 3–9 of *TANGO2* (12/22 alleles, 55%). Affected individuals were homozygous for the deletion in five families and compound heterozygous in four families. The remaining pathogenic variants in our cohort have not been previously reported and include a splice site variant (c.711-3C>G), a recurrent nonsense variant (c.94C>T, p.R32*, *n* = 3), a single-exon 6 deletion (arr[GRCh37] 22q11.21[chr22:20,042,250-20,048,850]), and two missense variants (c.77G>A, p.R26K; c.265G>T, p.G89C) (Fig. 4). We performed RNA expression studies of the c.711-3C>G variant using fibroblasts from patient 1, which showed that intron 8 is retained in the majority of transcripts (Fig. 2). This results in the insertion of 52 amino acids into the protein sequence before ending in a premature stop codon (NM_152906.6 p.R237ins*53). Occasionally, intron 7 is also retained in both control and patient transcripts (data not shown).

For most individuals, the *TANGO2* variants were identified by exome sequencing, often after prior metabolic and genetic testing. Patient 12 was first identified with a heterozygous 22q11 deletion detected by chromosome microarray during a workup for developmental delays. Additional episodic metabolic abnormalities including ketosis, hypoglycemia, and lactic acidosis prompted further analysis of *TANGO2*, which lies within the 22q11 deletion region. In two additional cases, *TANGO2-*related disorder was considered in the differential due to previous experience diagnosing an unrelated patient. In two families, the diagnosis was made upon reanalysis of exome data or after the diagnosis was made in a similarly affected younger sibling. In family 1, all three siblings share compound heterozygous variants in *TANGO2*. The oldest sibling, with a more severe course, also harbors a de novo variant of uncertain significance in *UBR5* (c.7554C>A, p.A2585E) that was not present in his siblings. A variant in *UBR5* has been previously associated with familial adult myoclonic epilepsy in one family.^4^

### Summary of clinical findings

#### Initial presentations

Onset of first symptoms ranged from 4 months to 27 months (Table 1, Table S3). The most frequent initial symptom was developmental delay (10/14). Patient 6 presented with a metabolic crisis and concurrent heart failure with dilated cardiomyopathy. Her sister (patient 5), presented at 18 months with speech delay and hypoglycemic generalized tonic–clonic seizures. In the setting of rhinovirus, patient 7 presented with a severe metabolic acidosis, hypoglycemia, hyperammonemia, and elevated creatine kinase at 4 months with acute to subacute left temporal and parietal cortical infarcts on brain magnetic resonance image (MRI). Other presentations include febrile seizures in patient 8 at 18 months with no episode of rhabdomyolysis until age 5 and an episode of ataxia and dizziness at age 2 in patient 10 with subsequent recurrent episodes of encephalopathy, hypoglycemia, and rhabdomyolysis. Patient 11 presented with global developmental delays at 6 months and subsequently developed spasticity and dystonia. His first severe episode of rhabdomyolysis did not occur until 13 years of life.

Phenotypically, individuals are nondysmorphic in appearance with the exception of wide-spaced teeth in patients 1 and 11 and hypodontia in patient 11 (Fig. 1b, c).

#### Neurologic findings

**Fig. 3:**
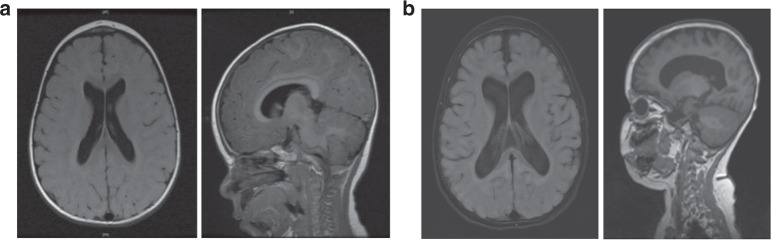
**Magnetic resonance imaging (MRI) of patient 1 demonstrating progressive cerebral atrophy.** **a** Axial T2 fluid-attenuated inversion recovery (FLAIR); sagittal T1 FLAIR at 9 months. **b** Axial 3D FLAIR; sagittal T1 at 21 months.

Seizures are present in 71% (10/14) of individuals, two of which were only related to episodes of hypoglycemia and another to ventricular tachycardia. Seizure types include infantile spasms, myoclonic, tonic, generalized tonic–clonic, partial complex, and multifocal seizures. Three individuals had refractory epilepsy. Brain MRI demonstrated cerebral atrophy in 5/14 patients. Cerebral infarct and/or cytotoxic edema during an acute episode was seen in 3/14 patients. Serial MR images from patient 1 highlight progressive generalized cerebral atrophy that occurred over the course of 1 year (Fig. 3). No structural brain abnormalities were seen in our cohort of patients.

Developmental delay was present in 86% (12/14) of individuals. This included gross motor delays with delayed walking in 5/14 patients. Walking was achieved between 10 months and 5 years. Language delays included first words between 18 months and 4 years. Moreover, when speech was acquired, it is described as difficult to understand or dysarthric. Significant regression occurred in 6/14, following acute metabolic decompensation or major illness. Subsequently, some individuals regained skills. IQ ranged from normal to severe ID when testing was performed. Muscle biopsy demonstrated nonspecific myopathic changes in 3/4 patients. Neurologic exam findings included variable muscle tone abnormalities including hypotonia, hypertonia, dystonia, and upper motor neuron signs including hyperreflexia, clonus, and positive Babinski. Lower extremities were more significantly affected with spastic Achilles tendons. Two patients required surgical intervention and/or botulinum toxin injections. In three individuals, multiple joint contractures were present. Progressive microcephaly was seen in 5/14 patients.

#### Ophthalmologic findings

Ophthalmologic findings were variable and included optic nerve atrophy, cortical vision impairment in 2/14. Patient 1 had the most severe ophthalmologic findings with abnormal conjugate, volitional eye movements with nystagmus, and progressive optic nerve atrophy. Additionally, 3/14 had abnormal eye movements consisting of amblyopia and dysconjugate gaze.

#### Laboratory findings

Documented hypoglycemia occurred in 9/14 individuals during acute metabolic crisis or severe illness. Ammonia levels were elevated in 3/14 patients with lactic acidosis present during metabolic crisis in 8/14 patients. Creatine kinase elevation was present in 11/14 patients ranging from 14,000 to 278,000 U/L with rhabdomyolysis documented in 9/14 patients. Elevation of transaminases was seen in 11/14 patients and was a direct result of rhabdomyolysis. Abnormalities of thyroid-stimulating hormone (TSH) and/or free T4 occurred in 7/14 patients with 5 patients receiving treatment with L-thyroxine.

#### Gastrointestinal findings

Of 14 patients, 8 had feeding difficulties requiring gastrostomy tube (GT) placements. Dysmotility was seen in 6/14 individuals with delayed gastric emptying studies. One patient required GT feeds only in the setting of decompensation.

#### Cardiac findings

Electrocardiogram abnormalities occurred in 6/14 individuals in the setting of metabolic crisis, including prolonged QTc, torsades de pointes, ventricular tachycardia and/or fibrillation. Cardiac arrest was seen in three individuals, one of whom had an implantable cardioverter-defibrillator (ICD). It is important to note that patients with severe arrhythmias were ultimately refractive to antiarrhythmia medication. Echocardiogram abnormalities included left ventricular hypertrophy, dilated cardiomyopathy, prominent trabeculations, and decreased left ventricular function when ill.

#### Causes of death

Of 14 individuals, 5 were deceased. Ages of death ranged from 8 months to 9.8 years. The time from initial symptoms to death ranged from 4 months to 9 years. The most frequent cause of death was cardiac arrest due to arrhythmias, which occurred in 4/5 deceased individuals. Arrhythmias at the time of death included uncontrolled ventricular tachycardia, long QT, and torsades de pointes. Only one individual had an ICD placed (patient 4). For patient 4, despite an ICD and antiarrhythmic medication during a hospitalization at 7 years 9 months, she died in the setting of uncontrollable sustained ventricular arrhythmias that was not responsive to antiarrhythmic medication. Patient 13 had ventricular tachycardia following her second metabolic crisis. It was initially responsive to flecainide; however, following 2 months of treatment she developed medication-resistant tachycardia and torsades de pointes causing her death. Patient 6 presented with a metabolic crisis, cardiomyopathy, and heart failure at 27 months that resolved; however, during a subsequent metabolic crisis at 3 years 10 months, she died due to sudden cardiac death. Patient 5, her sister, did not experience arrhythmias aside from her first metabolic crisis where she had dilated cardiomyopathy and long QT. She does not have an ICD and is not on antiarrhythmic medication. Her most recent 24-h Holter and echocardiogram have been normal. In family 1, where no arrhythmias have been documented, patient 1 died following initiation of comfort care after a significant course of epileptic encephalopathy, progressive cerebral and optic nerve atrophy, and visceral hyperalgesia.

Full case descriptions are provided in the Supplemental Material.

## Discussion

We report clinical and molecular results for 14 patients with *TANGO2*-related disease. Most of our patients experienced metabolic crises, rhabdomyolysis, seizures, and arrhythmias, which is consistent with previously described cases. However, we find that the initial presentation was more variable than previously reported, and some patients presented with primarily neurologic features. This suggests while the full *TANGO2*-related phenotype often emerges over time, many patients can present with initial features that are not suggestive of the final diagnosis. For example, patient 1 was initially thought to have an epileptic encephalopathy and then experienced neurodegeneration, while his brother (patient 2) presented with a milder encephalopathy with epilepsy; neither experienced rhabdomyolysis or life-threatening arrhythmias. Moreover, it is important to emphasize the heterogeneity in *TANGO2* as a progressive neurodegenerative disorder. We presented patients of different ages and functional states who are at different stages of the disorder. Variable expressivity is seen in families 1 and 3. In our series, significant decline during crises is apparent followed by full recovery, partial recovery, or overall loss of function. Moreover, progressive brain atrophy apparent in early to late stages of brain atrophy was a feature in 5/14 individuals.

**Fig. 4:**
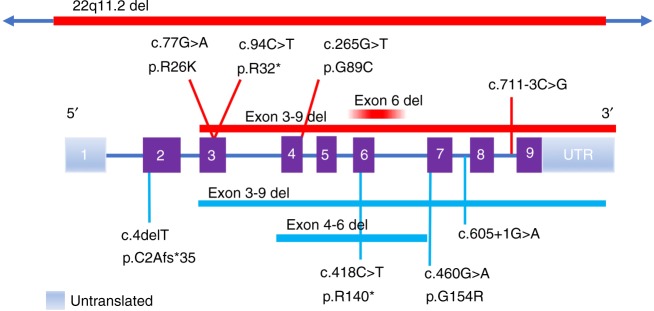
*TANGO2* gene illustration with a comparison of the molecular variants from the present study and previously described patients. Variants from the 14 patients described are shown above the gene depiction with red bars for deletions and lines for single nucleotide variants (SNV), and pathogenic variants from prior studies are shown below (light blue bars for deletions and lines for SNV) based on *TANGO2* transcript NM152906.6 (2623 bp).

Delayed or postmortem diagnosis occurred in a number of individuals, highlighting the importance of reanalysis of apparently negative ES as novel genes are identified as well as consideration of novel genes with atypical presentations.^5^ Moreover, the need to incorporate CNV and exon level deletion/duplication analysis is crucial as the intragenic deletion was missed with standard analysis of next-generation sequencing data in patient 12. Our results further illustrate the molecular heterogeneity seen in patients with *TANGO2*-related disease. Five of seven pathogenic variants identified in our cohort are novel, including splice site, missense, and nonsense variants as well as a single-exon deletion (Fig. 4).

An emerging theme in clinical genetics is that individuals can have two or more Mendelian disorders.^6,7^ If an individual’s clinical symptoms are not fully explained by their initial diagnosis, it is important to consider additional diagnoses. This was highlighted by patient 12, who had a known diagnosis of 22q11.2 deletion syndrome (22q11.2DS). Beyond what is expected in his primary diagnosis, he began experiencing episodic weakness and metabolic abnormalities. Given these new symptoms, further molecular analysis was sought, revealing the exon 3–9 deletion in *TANGO2*, which is within the region of recurrent microdeletions for 22q11.2DS. There were direct implications given his dual diagnosis because medical management is critically important during metabolic decompensation.

Molecular diagnosis of rare metabolic disorders can be challenging given their often extreme clinical and genetic heterogeneity. ES is a powerful diagnostic tool but is limited by current knowledge. Our cases illustrate the utility of ES data reanalysis because discovery of novel disease genes can diagnose previously unsolved cases. In addition, incorporating CNV analysis in ES data is imperative for *TANGO2*-related disorder. The potential for life-limiting medical complications such as metabolic crises and arrhythmias underscores the importance of early recognition and a rapid diagnosis of *TANGO2*-related disorder. The variability in initial presentation suggests that *TANGO2* should be considered in the genetic workup of developmental delay, childhood-onset seizures, rhabdomyolysis, and suspected neurologic or metabolic disorders. Early diagnosis is important so that providers and families are aware of potential metabolic crises associated with illness. Further work is necessary, specifically relating to the function of *TANGO2* and impact of specific variants on the function of the protein as this may lead to targeted therapies in affected individuals.

### URLs

The URLs for data presented include:

gnomAD, http://gnomad.broadinstitute.org/

UCSC Genome Browser, https://genome.ucsc.edu/

ExAC Browser, http://exac.broadinstitute.org/

## Electronic supplementary material

Supplemental Material
