# Correction: TANGO2: expanding the clinical phenotype and spectrum of pathogenic variants

**DOI:** 10.1038/s41436-018-0336-6

**Published:** 2018-10-15

**Authors:** Jennifer N. Dines, Katie Golden-Grant, Amy LaCroix, Alison M. Muir, Dianne Laboy Cintrón, Kirsty McWalter, Megan T. Cho, Angela Sun, J. Lawrence Merritt, Jenny Thies, Dmitriy Niyazov, Barbara Burton, Katherine Kim, Leah Fleming, Rachel Westman, Peter Karachunski, Joline Dalton, Alice Basinger, Can Ficicioglu, Ingo Helbig, Manuela Pendziwiat, Hiltrud Muhle, Katherine L. Helbig, Almuth Caliebe, René Santer, Kolja Becker, Sharon Suchy, Ganka Douglas, Francisca Millan, Amber Begtrup, Kristin G. Monaghan, Heather C. Mefford

**Affiliations:** 1 Department of Medicine, Division of Medical Genetics, University of Washington, Seattle, Washington USA; 2 Division of Genetic Medicine, Seattle Children’s Hospital, Seattle, Washington USA; 3 Department of Pediatrics, University of Washington, Seattle, Washington USA; 4 GeneDx, Gaithersburg, Maryland USA; 5 Division of Medical Genetics, Ochsner Health System and University of Queensland, Brisbane, Australia; 6 Ann & Robert H. Lurie Children’s Hospital of Chicago, Northwestern University Feinberg SOM, Chicago, Illinois USA; 7 Genetics and Metabolic Clinic, St. Luke’s Children’s Hospital System, Boise, Idaho USA; 8 Department of Neurology, University of Minnesota, Minneapolis, Minnesota USA; 9 Cook’s Children Genetic Center, Fort Worth, Texas USA; 10 Division of Neurology, Children’s Hospital of Philadelphia, Philadelphia, Pennsylvania USA; 11 Department of Neuropediatrics, Universitätsklinikum Schleswig Holstein Campus Kiel, Kiel, Germany; 12 Institute for Human Genetics, Universitätsklinikum Schleswig Holstein Campus Kiel, Kiel, Germany; 13 Department of Pediatrics, University Medical Center Eppendorf, Hamburg, Germany

Correction to: *Genetics In Medicine*; 10.1038/s41436-018-0137-y; online publication 24 September 2018

The original version of this Article contained an error in the spelling of the author J. Lawrence Merritt, which was incorrectly given as Lawrence Merritt. This has now been corrected in both the PDF and HTML versions of the Article.

