# Supplementary material for: TANGO2: expanding the clinical phenotype and spectrum of pathogenic variants
Source: Genet Med. 2018 Sep 24;21(3):601–7. doi: 10.1038/s41436-018-0137-y (PMC6752277; doi:10.1038/s41436-018-0137-y)
Supplement: Supplementary file 1 — Supplemental Material [file 41436_2018_137_MOESM1_ESM.docx]

**Supplemental Material**

Molecular:

Table S1 shows the minor allele frequency (MAF) for the missense and splice site variants seen in *TANGO2* in this study. For exon 3-9 deletion, the variable breakpoints reported in patients is likely due to the probe coverage of the specific platform but represent the same recurrent exon 3-9 deletion (NM_152906 (GRCh37): GeneDx: chr22(20,030,782-20,052,415); Ambry: chr22(20,008,631-20,053,447); Institut for Human Genetics: arr[GRCh37] 22q11.21(20,040,685-20,058,027)). This deletion has been reported in large population-based studies with a population minor allele frequency of 0.11% (3/2640 alleles) in white Europeans (Shaikh et al., 2009) and 0.06% (17/26,694 alleles) (Coe et al., 2014). The exon 6 deletion, detected by array CGH and exome sequencing, (NM_152906 (GRCh37): chr22(chr22:20,042,250-20,048,850)x0) has not been reported in control databases to our knowledge.

**Table S1.** Minor Allele Frequencies (MAF) of *TANGO2* variants

| MAF | c.77G>A  p.R26K | c.94C>T  p.R32* | c.265G>T  p.G89C | c.711-3C>G | Ex 6 Del | Ex 3-9 Del |
| --- | --- | --- | --- | --- | --- | --- |
| Total Population | 0% | 0.0029%  8/276,968 | 0.0006%  1/161,862 | 0.0016%  4/245,810 | U | 0.06%,  17/26,694 |
| European^a^ | 0% | 0.0016%  2/126,646 | 0.0015%  1/64,832 | 0.0036%  4/111,322 | U | ~0.062%  ≥11/17,644 |
| African | 0% | 0.0167%  4/24,030 | 0%  0/8,806 | 0%  0/15,304 | U | - |
| South Asian | 0% | 0.0033%  1/30,734 | 0%  0/23,544 | 0%  0/30,780 | U | - |

gnomAD Browser Accessed: 7/5/17

^a^European (non-Finnish, non-Ashkenazi); U=Unknown

The previously unreported splice site variant c.711-3C>G is highly conserved across species, has a high GERP score of 4.64, and is predicted to destroy the canonical splice acceptor site in intron 8. Abnormal splicing leading to an abnormal message that is subject to nonsense-mediated mRNA decay or an abnormal protein product is predicted to occur.

**Table S2:** Primer sequences used for RNA expression studies

| Target | Forward Primer | Reverse Primer |
| --- | --- | --- |
| *TANGO2:exon 5 – exon 9^* | 5’-CTCATAGCAGCCGACCTGAG-3’ | 5’-GTCCGCATCTACCAGGATGA-3’ |
| *TANGO2:exon 5 – intron 7^* | 5’-CTCATAGCAGCCGACCTGAG-3’ | 5’-CCTGGGTTTGCTGAGTGTCT-3’ |
| *GAPDH: exon 8 – exon 9* | 5’-CTTTGTCAAGCTCATTTCCTGG-3’ | 5’-TCTTCCTCTTGTGCTCTTGC-3’ |

^Transcript: NM_152906.6

Table S3. Extended summary of clinical findings of our patients in comparison to prior cases

|  | **P1** | **P2** | **P3** | **P4** | **P5** | **P6** | **P7** | **P8** | **P9** | **P10** | **P11** | **P12** | **P13** | **P14** | **Sum**  **(%)** | **Lit^1,2^**  **(%)** |
| --- | --- | --- | --- | --- | --- | --- | --- | --- | --- | --- | --- | --- | --- | --- | --- | --- |
| Pat. Allele | c.711-3C>G | c.711-3C>G | c.711-3C>G | Ex 3-9 del | c.94C>T  p.R32* | c.94C>T  p.R32* | c.265G>T  p.G89C | Ex 3-9 del | Ex 3-9 del | c.94C>T  p.R32* | Ex 3-9 del^ | 22q11.2 del | Ex 3-9 del^ | Ex 3-9 del | Fig 4 | Fig 4 |
| Mat. Allele | Ex 3-9 del | Ex 3-9 del | Ex 3-9 del | Ex 3-9 del | c.77G>A p.R26K | c.77G>A  p.R26K | Ex 3-9 del | Ex 3-9 del | Ex 3-9 del | c.94C>T  p.R32* |  | Ex 3-9 del |  | Ex 6 del | Fig 4 | Fig 4 |
| Family | 1 | 1 | 1 | 2 | 3 | 3 | 4 | 5 | 6 | 7 | 8 | 9 | 10 | 11 |  |  |
| Consanguin. | - | - | - | + | - | - | - | U | - | + | - | - | U | - | 2/14 (14) | 3/15 (20) |
| Ances-try | EU | EU | EU | EU, AA | AA | AA | EU | Latino | EU | Arab | Latino | EU | EU | EU |  |  |
| Age of Onset | 5m | 9m | 8m | 1yrs | 18m | 27m | 4m | 18m | 6m | 2yrs | 6m | 9m | 6m | 1yr | 4m-27m | 3.5m-8yr |
| Initial Symp | DD | DD | DD | DD | DD/  HG GTC | MC | MC | FS | DD | Ataxia | DD | DD | DD | DD | Table 1 | Table 1 |
| Age  at Dx | PM | 22m | 7m | 7yr2m | 4yr11m | PM | 5m | 26yr | 4yr8m | 7yr6m | 17yr | 3yr | 9yr7m | 2yr5m | 5m-17y |  |
| Present Age (d.) | d. 3yr | 3.5yr | 2yr | d. 7yr9m | 5yr | d. 3yr10m | d.8m | 26yr | 5yr | 7yrs | 17yr | 3yr8m | d. 9yr10m | 2yr11m | 8m-26yr | 11m-27yr |
| Laboratory Findings | | | | | | | | | | | | | | | | |
| HG | + | - | + | + | + | + | + | - | - | + | - | + | - | + | 9/14 (64) | 12/15 (80) |
| NH_3_ | N | N | N | N | N | N | ↑ | N | N | N | N | ↑ | ? | ↑ | 3/14 (20) | 9/15 (60) |
| Lactate | N | N | ↑ | ↑ | ↑ | ↑ | ↑ | N | N | N | - | ↑ | ↑ | ↑ | 8/14 (57) | 13/15 (87) |
| CK (U/L) | N | N | N | 14k | ↑ | ↑ | 105k | 50k | 88k | 217k | 278k | 896 | 2k | 64k | 11/14 (79) | 14/15 (93) |
| RM | ND | ND | ND | + | - | + | + | + | + | + | + | - | + | + | 9/14 (64) | 13/15 (87) |
| TSH/  Free T4 | ↑/N | N | N | ↑/N | N/↓ | ND | ND | ↑/↓ on tx | N | N | ↑/↓ on tx | ↑/↓ on tx | ↑/↓ on tx | ↑/↓ on tx | 8/14 (57) | 7/15 (47) |
| Gastrointestinal Manifestations | | | | | | | | | | | | | | | | |
| Feed.  Diff. | +, GT | +, GT | +, GT | - | -, GT | - | + | +, GT | - | +, GT | - | +, GT | - | +, GT | 9/14 (64) | 6/15 (40) |
| Dysmo-tility | + | + | + | - | - | - | - | + | - | + | - | + | - | - | 6/14 (43) | u |
| Cardiac Manifestations | | | | | | | | | | | | | | | | |
| Arrhy-thmia | - | - | - | +^g^ | +^h^ | - | +^i^ | - | - | - | - | +^j^ | +^c^ | - | 5/14 (36) | 10/15 (67) |
| SCA | - | - | - | + | - | + | - | - | - | - | - | - | + | - | 3/14 (21) | 2/15 (13) |
| Pace-maker | - | - | - | + | - | - | - | - | - | - | - | - | - | - | 1/14 (7) | 4/15 (27) |
| ECHO | LVH | ND | N | ↓ fx | DCM, ↓ fx | DCM | Mild ↓ fx | PT | N | N | N | N | N | N | 6/14 (43) | 2/15 (13) |
| Neurologic Findings | | | | | | | | | | | | | | | | |
| Seizures | + | + | + | + | + | - | + | + | - | + | - | - | + | + | 10/14 (71) | 12/15 (80) |
| Seizure Type  /EEG | Mult. Types/EE/SE^a^ | Tonic-clonic | Mult. Types/EE/SE^b^ | Partial Complex | HG GTC | n/a | Multi-focal, IS | GTC | n/a | HG GTC | EE | n/a | GTC | HG GTC, SE |  |  |
| Refrac-tory | + | - | + | - | - | n/a | + | - | n/a | - | n/a | n/a | - | - | 3/14 (21) | 1/3 (33) |
| Brain MRI | CA | N | ND | N | ND | ND | CI | CA, CI | PV | PV | N | ↓Vol.^b^ | N | CE w/ SE | 7/14 (50) | 10/15 (67) |
| Micro-cephaly | + | + | + | - | - | - | - | - | + | - | - | + | - | - | 5/14 (36) | 6/15 (40) |
| Neuro.  Findings | UMN/ dys. | LE hypo. | Truncal/LE hypo. | MHaH | N | N | - | SQ/C | MHaH | N | SQ/C | UMN/C | Hyper./  Hemi-paresis | UMN/  Hypo. | 10/14 (71) |  |
| DD | + | + | + | + | + | - | + | + | + | - | + | + | + | + | 12/14 (86) | 14/15 (93) |
| Speech  /age | - | +/18m | +/13m | +/2yr | +/18m | n/a | n/a | - | +/2yr, dysar. | + | +/4yr | - | +/2yr dysar. | - | 8/14 (57) |  |
| Walk-ing/age | - | +/ 21m | - | +/10m | +/1yr | n/a | n/a | +/w/a | +/25m | - | +/5yr, w/a | - | + | - | 7/14 (50) |  |
| Regres-sion | + | + | + | + | n/a | n/a | n/a | + | - | - | - | - | - | + | 6/14 (43) |  |
| ID | ND | ND | ND | ND | ND | - | ND | Sev. | ND | ND | Mod. | + | Sev. | Sev. | 5/14 (36) | 15/15 (100) |
| Ophthalmologic Findings | | | | | | | | | | | | | | | | |
| Ophth | Abn.^d^ | N | N | N | N | N | N | Abn.^e^ | p? | N | Abn.^f^ | N | N | N | 3/14 (21) | 2/15 (13) |

Additional details:

Patient 1 ^a^Infantile spasms, myoclonic, tonic, multifocal-bifrontal and right parietal; ^d^Optic atrophy, cortical visual impairment, abnormal movements, nystagmus;

Patient 3 ^b^Myoclonic, myoclonic-astatic, generalized tonic-clonic/EEG findings including epileptic encephalopathy and multifocal epileptiform discharges maximal in the bicentral region

Patient 4 ^g^ VT, VF, prolonged QTc ; Patient 5 ^h^prolong QTc;

Patient 6 ^i^wide complex VT, prolonged QTc; Patient 8 ^e^Dysconjugate gaze;

Patient 11 ^f^Mild cortical visual impairment; abnormal alignment, mild bilateral optic nerve pallor

Patient 12 ^b^posterior periventricular white matter, with minimal prominence of occipital horns of lateral ventricles; ^j^ rSR' pattern in the right-sided precordial leads

Patient 13 ^c^prolonged QTc, VT, TdP causing death

Abbreviations:

^=parental testing not done; “+”: Present; “-”: absent; AA: African American; Abn.: Abnormal; CK: Creatine Kinase, C: Contractures; d.: deceased (age at death); DD: Developmental Delay; DCM: Dilated Cardiomyopathy, Dysar.: Dysarthria; CA: Cerebral atrophy; CE: Cytotoxic edema, CI: Cortical infarct; EE: Encephalopathy; EEG: Electroencephalopgraphy; EU: European; Feed. Diff.: Feeding Difficulty; FS: Febrile Seizures; fx: Funtion, GT: Gastrostomy Tube; GTC: Generalized Tonic Clonic; HISP: Hispanic; HG: Hypoglycemia; Hyper.: Hypertonia; Hypo.: Hypotonia; HG GTC: Hypoglycemic Generalized Tonic Clonic; IS: Infantile spasms; ID: Intellectual disability; Lit (%): Summary of individuals from literature (Lalani et al; Kremer et al); LE: Lower Extremities, LVH: Left Ventricular Hypertrophy; Mat.: Maternal; MC: Metabolic Crisis; MHaH: Mixed Hypertonia and Hypotonia; n/a: not applicable; Neuro. Find.: Neurologic Findings; N: normal; ND: Not Done; P#: Patient #; Pat: Paternal; PM: Post-Mortem; PT: Prominent Trabeculations, PV: Prominent ventricles; RM: Rhabdomyolysis, SQ: Spastic Quadraplegia; Sum (%): Summary of individuals from our case series; SE: Status Epilepticus; TdP: Torsades de Pointes, U: Unknown; UMN: Upper motor neuron signs present; VF: Ventricular Fibrillation, VT: Ventricular Tachycardia; w/a: with assistance

Clinical Information

Family 1 involves three siblings with compound heterozygous *TANGO2* variants who presented with a clinically distinct phenotype from previously reported patients with primarily neurologic features, progressive microcephaly, failure to thrive requiring gastrostomy tubes (G-tubes) and one severely affected sibling with epileptic encephalopathy, neurodegeneration, optic atrophy, and visceral analgesia. Their parents are non-consanguineous and of European descent. Following the death of the eldest sibling, a quad exome at GeneDx identified compound heterozygous, rare deleterious variants in *TANGO2*. A maternally inherited deletion involving exons 3-9 of *TANGO2* and c.711-3C>G, a splice site variant that is predicted alter splicing.

The older sibling, Patient 1, presented with global DD and progressive microcephaly that began at 5 months. He had dysphagia with failure to thrive by 6 months and was G-tube dependent by 8 months. He developed increased tone and reflexes, posturing episodes and ocular motility disorder. Infantile spasms with modified hypsarrhythmia were noted by 1 year and treated with ACTH. Additional seizure types included myoclonic and tonic upper extremity stiffening with eye movement deviation and generalized seizures. Seizure activity increased during illness and was refractory to multiple medications. An EEG at 14 months showed interictal epileptiform abnormalities and left frontotemporal spikes. Myoclonic status epilepticus, generalized tonic seizures, and epileptic spasms were seen at 20 months. Brain MRI at 9, 15 and 21 months showed progressive cerebral atrophy and thinning of the corpus callosum. MR spectroscopy demonstrated a lactate peak in the basal ganglia and CSF. Echocardiogram at 14 months revealed left ventricular hypertrophy; EKG was normal. Abnormal eye movements with nystagmus progressed, and he developed cortical visual impairment and optic nerve atrophy. He had multiple hospitalizations, often characterized by vomiting, abdominal pain and distention, agitation, hypertension, and dystonia which were attributed to visceral hyperalgesia. In addition, he had hospitalizations for status epilepticus and increased seizure frequency with no clear infectious source. He had nephrolithiasis, possibly due to zonisamide treatment. Muscle biopsy was nonspecific with myopathic changes and low-normal complex I activity. An extensive genetic and biochemical workup culminated in a nondiagnostic trio ES. Ultimately, the family sought comfort care and he died at the age of 3.5 yrs.

Patient 2 had a milder presentation that also included progressive microcephaly, global DD, and epilepsy. He developed generalized tonic-clonic seizures at 11 months that are well controlled as well as brief atonic head drops. Brain MRI at 1 year was unremarkable; spectroscopy showed a lactate peak in the CSF. He achieved walking at 21 months and lacks pyramidal signs that were present in his brother at the same age. He experiences mild regression during illness but has not required hospitalization. Similar to Patient 1, he has hypercalciuria and increased excretion of oxalate, but he has no overt nephrolithiasis. He had a normal EKG and VEP at 18 months. Due to feeding intolerance, he received a GT at age 2. By age 3, his seizures continued to be well-controlled and his AED medication was weaned at age 3.5 (2 years seizure-free). At 3.5 years, his receptive language is developmentally appropriate. For expressive language, he has 20 words and knows an additional 20 signs. His gait continues to be wide based and mildly uncoordinated and he is able to run. ES revealed compound heterozygous variants in *TANGO2*: a previously published maternally inherited intragenic deletion of exons 3-9 and a paternally inherited rare c.711-3C>G (IVS8-3C>G) splice variant. The same variants were found in his brother’s exome upon reanalysis.

Targeted testing confirmed compound heterozygote variants in *TANGO2* in Patient 3. Clinically, she has difficulty with PO feeding and delayed gastric emptying requiring G-tube placement at 10 months. Consistent with her brothers, her head circumference was 50% at birth and 12% at 9months. An ECHO and ECG were both normal at 9 months. She developed myoclonic seizures at 15 months. At age 20 months, she was hospitalized for a parainfluenza and rhinovirus-enterovirus illness. During the admission, she developed Enterobacter bacteremia and experienced hypoglycemia to 54mg/dL, and mild lactic acidosis to 2.9mmol/L (NR 0.5 to 2.2). Her CK and ammonia levels were normal and had no evidence of rhabdomyolysis in her urine. At 2 years, EEG demonstrates epileptic encephalopathy and multifocal epileptiform discharges maximal in the bicentral regions seizures. Additionally, she has additional seizure types including myoclonic-astatic and generalized tonic-clonic seizures. Unlike her two siblings, she did not respond to zonisamide and is currently on lamotrigine, topiramate, and levotiracetam. Prior to this developmentally, she had mild delays with intervention including physical and occupational therapy. At 18 months, she was cruising, taking independent steps, and crawling. At 20 months, she experienced regression following illness and is now unable to take steps or cruise, and her head control has worsened. She has 20 words.

Patient 4, from Family 2, presented at 1 year with developmental delay, specifically speech delay with her first words at 2 years. She regressed with febrile seizures at the age of 2, and developed complex partial seizures Her parents were first cousins and of European and African American ancestry. She was homozygous for exon 3-9 deletion. She had intellectual disability with a normal MRI and MRA. Her TSH was elevated with normal free T4 levels. She experienced episodic metabolic abnormalities including an elevated CK-MB of 14,000U/L with rhabdomyolysis, mildly elevated AST, ALT, lactate of 4.2mmol/L, hypoglycemia with lowest recorded of 46mg/dL. On muscle biopsy, she had a complex IV deficiency. Given repeated episodes of ventricular tachycardia and long QT she required an implantable cardioverter defibrillator (ICD). At 7yr2m, she had normal growth parameters, no difficulty with feeding, and her ophthalmologic exam was normal. Neurologic exam demonstrates mild hypotonia with diminished reflexes, diffuse muscle weakness that is more pronounced in her lower extremities, mild dysmetria, and spasticity of her Achilles tendons. At 7yr9m, she was hospitalized in the ICU. Her admission ventricular function was 54% with normal right ventricular function. It declined and she received multiple shocks and defibrillations from her AICD following episodes of ventricular tachycardia and ventricular fibrillation. During the hospitalization, she had significant rhabdomyolysis (18,000U/L), and she died in the setting of uncontrollable sustained ventricular arrhythmias.

From family 3, parents are non-consanguineous and of African American descent. Patient 6 initially presented with elevated CK levels, rhabdomyolysis, hypoglycemia and dilated cardiomyopathy with heart failure at 27 months. With illness, she exhibited hypoglycemia, elevated transaminases and lactic acid, and poor oral intake. Her growth parameters, development and neurologic exam were all reportedly normal. Acylcarnitine profile was consistent with very long-chain acyl-CoA dehydrogenase deficiency (VLCADD) but no variants were identified on molecular studies. She was placed on fat restricted diet using metabolic formula and MCT supplementation. Ultimately, she died at 3 years 10 months of life due to sudden cardiac death during a metabolic crisis. Her younger sister, Patient 5, presented at 18 months of age with delayed speech and hypoglycemic generalized tonic clonic seizures. She walked at 1 year. Similar to her sister, she had episodes of metabolic decompensation in the setting of illness with decreased PO intake, hypoglycemia, mildly elevated lactic acid and transaminases, elevated CK, and generalized tonic-clonic seizures. Her ammonia levels remained normal during metabolic decompensation with no evidence of rhabdomyolysis. One severe episode resulted in cardiac failure with dilated cardiomyopathy on echocardiogram. EKG showed prolonged QTc and severely depressed left ventricular function. This has since normalized and her cardiac function returned to baseline with no history of cardiac arrest or pacemaker placement. She is currently on an ACE inhibitor and a VLCADD-like diet. Due to the severity of the episode and poor PO intake post crisis, a G-tube was placed. Outside of illness or triggered episodes, only the formula is given via G-tube. She is currently 5 years old and has normal growth parameters, no abnormalities on neurologic exam including a normal gait. She has a low free T4 with normal TSH. Her speech is dysarthric, and is mainly single words with a few short phrases. Receptive language appears more appropriate for age, and she is able to follow multistep commands. Patient 5 was tested with exome trio and subsequent targeted testing of her deceased sister revealed that she was also a compound heterozygote for *TANGO2*.

Patient 7 was born to non-consanguineous parents of European descent. He presented at 4 months of life with rhinovirus and experienced an acute metabolic decompensation with hypoglycemia, acidosis, rhabomyolysis, CK elevations (peak of 104,527U/L), and elevated transaminases with a small acute to subacute left temporal and parietal cortical infarcts. Initially, he had a pH of 7.14 with a glucose level that was undetectable. His lactic acid level was 5.5mmol/L. Moreover, he had wide complex ventricular tachycardia and prolong QTc. Echocardiogram demonstrated a mild decrease in function when acutely ill. With his first episode, his AST was 2327IU/L and ALT was 449IU/L. Seizures were multifocal (central parietal and right frontal central, left parietal and left temporal) and refractory to multiple AEDs including levetiracetam, lacosamide, and clonazepam. Subsequently, he developed infantile spasms by 6 months, could not lift his shoulders up, and had significant head lag of 4-5 seconds. At 8 months of life, he died during his third metabolic crisis with a peak ammonia level of 111mcmol/L.

Patient 8 is a Latino female with homozygous exon 3-9 deletion. Further details about her parents are unknown as she was adopted. She presented with generalize tonic-clonic (GTC) seizure associated with fever which was initially diagnosed as febrile at 18 months. This episode was followed by regression, including the loss of crawling, speech, and the need for assistance when walking. She improved but never returned to her previous baseline. She was noted to have a unilateral polycystic kidney and underwent resection between her 1st and 2nd year of life. Following resection, her hypertension resolved. She has hypothyroidism on replacement. Her first episode of rhabdomyolysis was at age 5 years and was associated with GTC. This was followed by a second at age 17, and third at 22 years presenting in status epilepticus. During a metabolic crisis, brain MRI showed ventriculomegaly, diffuse atrophy of her right hemisphere greater than the left. There was restricted diffusion in the left parietal and occipital lobes mainly involving white matter. She has elevated CK, with the highest at age 23 of 50,170IU/L. EMG was normal at age 24 y. A muscle biopsy showed non-specific myopathic changes, and normal electron transport chain analysis. Cardiac MRI showed normal left ventricular size and function with prominent trabeculations. She has had no documented episodes of hypoglycemia as well as normal ammonia and lactate levels. On exam, she had disconjugated gaze; abnormal muscle tone of mixed type with limited range of purposeful movements including slow upper extremities and minimal to no movements in her lower extremities with contractures in her knees, ankles and elbows. For her vision, external, slit lamp and dilated eye exams are normal. She has mild myopic astigmatism. For mobility, she crawled until age 26; however, following a prolonged hospitalization she lost the ability to do so. For nutrition, she has a G-tube in place. She has severe intellectual disability and is nonverbal. She remains on AEDs with seizures that are well-controlled

Patient 9 was born to non-consanguineous European parents. Developmental delay was first recognized at 6 months. She was diagnosed at 4 years of life. She first sat at 12 months, walked at 25 months, first words were at 2 years old, and has ~ 10 words at 4 years. On MRI, she has mild prominence of the supratentorial ventricular system. She has had one episode of metabolic decompensation with CK of 88,000IU/L, with rhabdomyolysis, elevated transaminases, a normal ammonia, glucose, and lactate levels. She has microcephaly (head circumference 2^nd^ percentile, weight 53rd percentile, and height 32nd percentile). On neurologic exam, she has upper motor neuron signs including hypertonicity in her lower extremities, upward going toes, mild axial hypotonia, dysarthria, and ataxic gait. She is able to walk with support. Her TSH and free thyroxine were both normal.

Patient 10 presented at 2 years of age with episodes of dizziness and ataxia. His parents are second-degree cousins and are of Arabic descent. He has a history of recurrent metabolic decompensation with encephalopathy, rhabdomyolysis, renal/liver dysfunction, and hypoglycemic seizures. His highest CK level has been 217,000IU/L. During a metabolic crisis, he has normal ammonia and lactate levels and no associated, arrhythmias. Brain MRI showed mild prominence of the ventricles and sulci. A muscle biopsy demonstrated denervation atrophy with reinnervation. At the age of 7, his growth parameters, neurologic exam, development, and gait are normal. Due to poor feeding, gastrointestinal dysmotility, and constipation, he is G-tube fed. He has a murmur on exam; however, ECHO done in 2014 and 2017 were both normal.

Patient 11 presented with global developmental delay at 6 months. He was born to non-consanguineous parents of Latino ethnicity. He sat at 8 months, crawled at 1 year, walked at 5 years, and first words were at 4 years. By the age two, he received botox injections for spasticity. At 3 years of life he had episodes of weakness, dystonia, and ptosis. EEGs did not show epileptiform activity. By age 10, he had primary hypothyroidism and is currently on levothyroxine. At 13 years, he was hospitalized for a severe episode of rhabdomyolysis. His CK peaked to 278,000IU/L with elevated transaminases. During this and other metabolic episodes, his ammonia and glucose levels have been normal. At 15-16 years, he had Achilles tendon and hamstring release due to significant spasticity. On SNP microarray, there were regions of homozygosity on chromosome 22, including *TANGO2*. A custom gene panel was sent, which included *TANGO2* and subsequently, he was found to be homozygous for the exon 3-9 deletion at age 17. His EEGs are consistent with encephalopathy and demonstrate no seizures; he has a normal brain MRI. At age 17, he has moderate intellectual disability. He can put two words together with dysarthric speech. Ophthalmologic evaluation demonstrated mild cortical visual impairment, amblyopia of the left eye, large angle esotropia bilaterally. ECHO and EKG are normal. For his growth, his weight is -2.75 SD, height is -2.1 SD and OFC is in the 25^th^ percentile. On exam, he has significant spasticity in his upper and lower extremities. Deep tendon reflexes are brisk and he has intermittent clonus. His gait is impaired by his spasticity; however, can take steps with assistance. His teeth are widely spaced, canonical in shape, with hypodontia including absent lateral maxillary incisors (see Figure 1C for photos). He is able to take all his calories by mouth and has occasional dysphagia with significant ptyalism.

Patient 12 was born to non-consanguineous parents of European descent. He presented with developmental delay and first sat at 9 months, crawled at 15 months; at 3 years he is nonverbal and cannot walk independently. A microarray was done for developmental delay and he was found to carry a deletion of 22q11.2. His symptomatology was outside of expectations for 22q11.2 deletion syndrome, with his first episode of muscle weakness at 17 months. Moreover, he has recurrent hypoglycemia, ketosis, lactic acidosis and elevated ammonia. ES was sent due to a suspicion for a mitochondrial disorder. No alleles amplified for *TANGO2*. Reanalysis of the ES data (Ambry) identified the exon 3-9 deletion, confirming a compound heterozygous deletion of *TANGO2*, diagnosed at age 3. On brain MRI, he has white matter loss in the periventricular region with minimal prominence of the occipital horns of the lateral ventricles. He does not have seizures and has a normal ophthalmologic exam. His CK level maximum was 896U/L with no rhabdomyolysis. An ECHO was normal and electrocardiogram showed a rSR' pattern in the right-sided precordial leads suggestive of an incomplete right ventricular conduction delay with a normal QTc (439 msec). AST/ALT is elevated to 337U/L and 276 U/L respectively. His ammonia levels range from 8.7-115microM and has chronic elevation of lactate ranging from 2.7 to 11mM. He is in the 3^rd^ percentile for weight, 2^nd^ percentile for height, and his head circumference is less than the 1^st^ percentile. On exam, he has increased tone in his lower extremities requiring botox injections, upward going toes, and hypotonia requiring hand splints. A G-tube was placed due to feeding issues.

Patient 13 presented with developmental delay at 6 months. She is of European ancestry and the relationship of her parents is unknown. By 1 year she had motor and speech delay. By age 8, she could speak in short sentences with dysarthria. At that time, she was suspected of having seizures; however, EEG was normal. In the context of a crisis she had tachycardia, cyanosis and apnea. In addition, she had intermittent hemiparesis with a typical duration of <30minutes. Following her first episode of decompensation, she had enuresis and encopresis at age 9. In the setting of viral infections, she had a total of 3 crises with initial symptoms of muscle pain and weakness, refusing to walk, elevated CK with a maximum of 2143 U/l, elevated serum myoglobin to 1520ng/ml (range<70ng/ml) and urine myoglobin 704microgram/mml (RR<2), elevated transaminases, lactic acidosis, without hypoglycemia. Muscle MR during rhabdomyolysis showed hyperintensities compatible with myositis. Diagnosis was made at 9yr7m by array CGH. At 9yr10m, she had a single generalized tonic-clonic seizure during an episode of ventricular tachycardia. Brain MRI and ECHO were both normal. On EKG, she had prolonged QTc to >600ms with severe arrhythmias including ventricular tachycardia. She was treated with Flecainide therapy that two months later was therapy resistant. She subsequently died following Torsade-de-Pointes tachycardia. TSH was 10.4 ulU/l (RR 0.6-4.84) and was acutely elevated during crisis to 37.5uIU/ml with normal TPO-AK. She was treated with levothyroxin treatment. For her vision, she has normal fixation and no apparent abnormalities. On neurologic exam, she was hypotonic; however, did attain independent ambulation. She had severe intellectual disability.

Patient 14 presented at 1 year with global developmental delay; at 23 months, she was unable to walk with no speech. She was born to non-consanguineous parents of German descent. During acute crisis she experienced hypoglycemia, elevated CK (max of 64,000U/l), elevated transaminases, elevated ammonia, lactic acidosis, and severe rhabdomyolysis. Moreover, she has generalized tonic-clonic seizures related to hypogylcemia and a history of status epilepticus. A brain MRI at 16 months was normal; however, in the context of status epilepticus, brain MRI demonstrated cytotoxic edema. Her seizures are treated with phenytoin and were refractive to levetiracetam. At 2year 4months she had a severe deterioration and regression after an encephalopathic crisis including the loss of the ability to stand and feeding difficulties requiring G-tube feeds when prior she ate without difficulties. TSH was elevated to 16.7mIU/L with a free T3 of 1.69pmol/l (RR 3.69-8.46). TPO-AK was normal and she is treated with levothyroxin. For her vision, she has normal fixation and no apparent abnormalities. EKG and ECHO were both normal with no history of arrhythmias. On exam, she has spastic paraplegia, positive Babinski sign, and generalized hypotonia. By 2.5 years she was diagnosed with *TANGO2*-related disorder on array.
